# Profile and Usefulness of Serum Cytokines to Predict Prognosis in Myelin Oligodendrocyte Glycoprotein Antibody−Associated Disease

**DOI:** 10.1212/NXI.0000000000200362

**Published:** 2025-01-03

**Authors:** Javier Villacieros-Álvarez, Carmen Espejo, Georgina Arrambide, Alessandro Dinoto, Patricia Mulero, Laura Rubio-Flores, Pablo Nieto, Carmen Alcalá, Jose E. Meca-Lallana, Jorge Millan-Pascual, Pedro Martínez-García, Raphael Bernard-Valnet, Inés González-Suárez, Aída Orviz, Raquel Téllez, Laura Navarro Cantó, Silvia Presas-Rodríguez, Sergio Martínez-Yélamos, Juan Pablo Cuello, Ana Alonso, Raquel Piñar Morales, Gary Álvarez Bravo, Lakhdar Benyahya, Sophie Trouillet-Assant, Virginie Dyon-Tafan, Caroline Froment Tilikete, Aurélie Ruet, Bertrand Bourre, Romain Deschamps, Caroline Papeix, Elisabeth Maillart, Philippe Kerschen, Xavier Ayrignac, Àlex Rovira, Cristina Auger, Bertrand Audoin, Xavier Montalban, Mar Tintore, Sara Mariotto, Alvaro Cobo-Calvo, Romain Marignier

**Affiliations:** 1 Neurology Department, Centre d'Esclerosi Múltiple de Catalunya (Cemcat), Hospital Universitari Vall d'Hebron, Vall d'Hebron Institut de Recerca;; 2 Universitat Autònoma de Barcelona, Spain;; 3 Neurology Unit, Department of Neurosciences, Biomedicine, and Movement Sciences, University of Verona, Verona, Italy;; 4 Servicio de Neurología. Hospital Clínico Universitario de Valladolid;; 5 Synaptia Madrid Neurosciences, Vithas La Milagrosa, Aravaca & Arturo Soria University Hospitals, Madrid, Spain;; 6 Servicio de Neurología, Hospital Universitario Rey Juan Carlos, Madrid, Spain;; 7 Unidad de Neuroinmunología, Hospital Universitari i Politècnic La Fe;; 8 Multiple Sclerosis CSUR. Clinical Neuroimmunology Unit, Neurology Department, “Virgen de la Arrixaca” Clinical University Hospital, IMIB-Arrixaca, and NICEM Cathedra, UCAM-San Antonio Catholic University, Murcia, Spain;; 9 Immunology Department, “Virgen de la Arrixaca” Clinical University Hospital, IMIB-Arrixaca, Murcia, Spain;; 10 Service of Neurology, Department of Clinical Neurosciences, Lausanne University Hospital (Centre Hospitalier Universitaire Vaudois) and University of Lausanne, Switzerland;; 11 Hospital Álvaro Cunqueiro, Vigo;; 12 Hospital Universitario Fundación Jiménez Díaz, Madrid;; 13 Neurología. Hospital General Universitario de Elche, Alicante;; 14 MS-Neuroimmunology Unit, Neurosciences Department, Germans Trias i Pujol Hospital;; 15 Multiple Sclerosis Unit, Department of Neurology, Hospital Universitari de Bellvitge, Neurology and Neurogenetics Group, Neuroscience Program, Department of Clinical Sciences, Institut d'Investigació Biomèdica de Bellvitge (IDIBELL);; 16 Hospital General Universitario Gregorio Marañón;; 17 Hospital Regional Universitario de Málaga;; 18 Servicio de Neurologia. Hospital Universitario Clinico San Cecilio, Granada, Spain;; 19 Unitat de Neuroimmunologia i Esclerosi Múltiple Territorial Girona (UNIEMTG), Hospital Universitari de Girona Dr. Josep Trueta | Hospital Santa Caterina, Girona, Spain;; 20 Hospices Civils de Lyon, Service de Neurologie, Sclérose en Plaques, Pathologies de la Myéline et Neuro-Inflammation-Hôpital Neurologique Pierre Wertheimer, Bron Cedex;; 21 Joint Research Unit Hospices Civils de Lyon, Hôpital Lyon Sud, Pierre-Bénite;; 22 Hospices Civils de Lyon, Service de Neuro-Ophtalmologie-Hôpital Neurologique Pierre Wertheimer, Bron Cedex;; 23 Centre Hospitalier Universitaire de Bordeaux;; 24 CHU-Hôpitaux de Rouen;; 25 Foundation Adolphe de Rothschild Hospital;; 26 Department of Neurology, AP-HP. Hôpital Pitié-Salpêtrière; Centre de Référence des Maladies Inflammatoires Rares du Cerveau et de la Moelle (MIRCEM), Paris;; 27 Centre Hospitalier de Luxembourg, Luxembourg-Ville;; 28 Montpellier University Hospital;; 29 Section of Neuroradiology, Department of Radiology (IDI), Hospital Universitari Vall d'Hebron, Vall d'Hebron Institut de Recerca, Universitat Autònoma de Barcelona;; 30 Aix Marseille Univ, APHM, Hôpital de la Timone, CNRS, CRMBM, Marseille, France; and; 31 Universitat de Vic, Spain.

## Abstract

**Objectives:**

To characterize the serum cytokine profile in myelin oligodendrocyte glycoprotein antibody–associated disease (MOGAD) at onset and during follow-up and assess their utility for predicting relapses and disability.

**Methods:**

This retrospective multicentric cohort study included patients aged 16 years and older meeting MOGAD 2023 criteria, with serum samples collected at baseline (≤3 months from disease onset) and follow-up (≥6 months from the baseline), and age-matched and time to sampling–matched patients with multiple sclerosis (MS). Eleven cytokines were assessed using the ELLA system. Data comparisons and statistical analyses between cytokine levels and clinical outcomes were performed.

**Results:**

Eighty-eight patients with MOGAD and 32 patients with MS were included. Patients with MOGAD showed higher IL6 (*p* = 0.036), IL8 (*p* = 0.012), and IL18 (*p* = 0.026) baseline levels compared with those with MS, in non–optic neuritis (ON) presentations. BAFF values increased over time, especially in patients with MOGAD treated with anti-CD20 (*p* = 0.002). Baseline BAFF, CXCL10, IL10, and IL8 levels correlated with disease severity at MOGAD onset (all *p* < 0.05). Finally, higher baseline BAFF levels predicted lower risk of relapses (hazard ratio 0.41 [0.19; 0.89], *p* = 0.024).

**Discussion:**

This study suggests a proinflammatory Th17-dominant profile in non-ON MOGAD patients, with a novel finding of a potential protective role of BAFF on relapses. These results shed new light on the pathogenesis of MOGAD, potentially guiding therapeutic decisions.

## Introduction

Similar to other antibody-mediated conditions, the autoimmune process of myelin oligodendrocyte glycoprotein antibody–associated disease (MOGAD) presumably initiates in the periphery.^1^ The study of cytokines in serum can help unravel the different pathways and immune cell types involved in the pathogenesis of this disease. It is important to note that they may lead to the discovery of effective therapies as reported with the humanized IL6-receptor antibody, satralizumab, in aquaporin-4 antibody-positive neuromyelitis optica spectrum disorder (AQP4-NMOSD)^2^ and serve as prognostic biomarkers.

In MOGAD, Th17 (IL6, IL8), Treg (IL10), and B-cell–related (BAFF, APRIL, BLC/CXCL13, CCL19) cytokines are upregulated in both pediatric and adult patients.^3-5^ However, most studies were conducted in CSF, with small sample sizes and without a prespecified protocol of sample collection. Moreover, robust data on the usefulness of these proteins for prognosis are lacking.

Therefore, our aims were to (1) characterize the serum cytokine profile in patients with MOGAD at onset and during follow-up and (2) assess the usefulness of these cytokines for predicting relapses and disability in MOGAD.

## Methods

This is a retrospective multicentric cohort study including patients aged 16 years and older fulfilling MOGAD 2023 criteria,^6^ with available serum samples obtained at baseline (≤3 months from disease onset) and follow-up (≥6 months from the baseline sample). Age-matched and time to first sampling–matched patients with MS were included as controls. Demographic and clinicoradiologic data at onset and during follow-up were collected.

A panel of 11 cytokines (eTable 1) was assessed in serum using the automated microfluidic analyzer ELLA (BioTechne, Minneapolis, MN) (eMethods). IL12p70 and IL17A were detected in <10% of the patients and were not included in statistical analyses.

Statistical analyses are described in the eMethods.

The study was approved by the Clinical Research Ethics Committee at Vall d’Hebron University Hospital (EPA [AG]57/2013 [3834]) and French ethical committee (Comité de Protection des Personnes [CPP]: reference 2019-A03066-51). All patients signed written informed consents.

Anonymized data not published within this article will be made available by request from any qualified investigator.

## Results

Eighty-eight patients with MOGAD and 32 patients with MS were included. Detailed baseline and follow-up patient characteristics are provided in Table 1.

**Table 1:** Demographic and Clinical Characteristics

Baseline characteristics	Whole cohort (n = 120)	MOGAD (n = 88)	MS (n = 32)	*p* Value
Female; no. (%)	73 (60.3)	47 (53.4)	25 (78.1)	0.026
Age at onset; y, median (IQR)	36.2 (27.8–46.2)	36.6 (27.4–50.5)	35.8 (28.1–41.4)	0.211
Topography at onset; no. (%)				
Optic nerve	58 (48.3)	45 (51.1)	13 (40.6)	0.417
Spinal cord	39 (32.5)	28 (31.8)	11 (34.4)	0.965
Encephalic	5 (4.2)	5 (5.7)	0 (0.0)	0.389
Other	18 (15.0)	10 (11.4)	8 (25.0)	0.119
EDSS score at onset; median (IQR)	2.25 [1.0–3.1]	2.75 [1.5–4.0]	2.00 [1.0–3.0]	0.045
CSF-OBs; no. (%)	41/98 (41.8)	16/68 (23.5)	25/30 (83.3)	<0.001
Time from onset to first MRI, median (IQR), d	12 (3–117)	9 (2–18)	97 (19–117)	<0.001
No. of brain lesions, n (%)				<0.001
0 lesions	39/111 (35.1)	39/79 (49.4)	0 (0.0)	
1–8 lesions	34/111 (30.6)	26/79 (32.9)	8/32 (25)	
≥9 lesions	38/111 (34.2)	14/79 (17.7)	24/32 (75)	
No. of brain CELs, n (%)				<0.001
0 lesions	81/109 (74.3)	69/83 (83.1)	12/26 (46.2)	
≥1 lesion	28/109 (25.7)	14/83 (16.9)	14/26 (53.9)	
No. of spinal lesions, n (%)				0.024
0 lesions	39/69 (56.5)	30/45 (66.7)	9/24 (37.5)	
≥1 lesion	30/69 (43.5)	15/45 (33.3)	15/24 (62.5)	
Time from disease onset to first sampling; mo, median (IQR)	0.8 (0.3–1.6)	0.7 (0.2–1.9)	0.9 (0.7–1.5)	0.414
Time from disease onset to second sampling; mo, median (IQR)	10.2 (7.6–20.8)	9.9 (7.1–16.8)	49.1 (10.2–96.6)	0.001
Time between first and second sampling; mo, median (IQR)	9.2 (6.3–19.5)	8.9 (6.0–15.5)	48.0 (9.5–95.2)	0.001
Acute treatment within one-month before baseline sampling; n (%)	61 (50.8)	47 (53.4)	14 (43.8)	0.446
Acute treatment within one-month before second sampling; n (%)	7 (5.8)	7 (7.9)	0 (0.0)	0.187
Disease duration; y, median (IQR)	4.3 (2.3–7.8)	3.0 (1.9–6.2)	8.8 (6.5–13.8)	<0.001
Follow-up characteristics				
Follow-up; y, median (IQR)	2.5 (0.8–7.0)	1.8 (0.8–4.4)	7.5 (4.7–11.4)	<0.001
Chronic treatment during follow-up*; no. (%)	69 (57.5)	48 (54.5)	21 (65.6)	0.443
Patients under chronic treatment at first sampling; no. (%)	6/69 (8.7)	6/48 (12.5)	0/21 (0.0)	0.094
Patients under chronic treatment at second sampling; no. (%)	54/69 (78.3)	37/48 (77.1)	17/21 (81.0)	1.000
Patients relapsing; no. (%)	41 (34.7)	28 (31.8)	14 (43.8)	0.320
Time to second relapse; wk, median (IQR)	20.2 (9.7–68.4)	15.3 (6.8–37.9)	44.3 (19.6–132)	0.016
EDSS score at the last follow-up; median (IQR)	1.00(0.00–2.00)	1.00(0.00–2.00)	1.25(1.00–2.00)	0.046
EDSS score ≥3.0 at the last follow-up; no (%)	18 (15.0)	13 (14.8)	5 (15.6)	1.000

Abbreviations: CELs = contrast-enhancing lesions; CSF-OBs = cerebrospinal fluid–restricted oligoclonal bands; EDSS = Expanded Disability Status Scale; IQR = interquartile range; MOGAD = myelin oligodendrocyte glycoprotein antibody–associated disease.

Additional missing values: EDSS scores at onset, n = 8 in the MOGAD cohort.

*In patients with MOGAD, the number of chronic treatments included the following: anti-CD20, 19; azathioprine, 11; mycophenolate mofetil, 5; prednisone, 8; IV immunoglobulin, 1; interferon, 2; glatiramer acetate, 1; cladribine, 1; teriflunomide, 1.

*In patients with MS, chronic treatments included the following: alemtuzumab, 1; cladribine, 1; dimethyl fumarate, 1; fingolimod, 1; interferon, 3; glatiramer acetate, 11; clinical trial, 1; natalizumab, 1.

**Figure 1:**
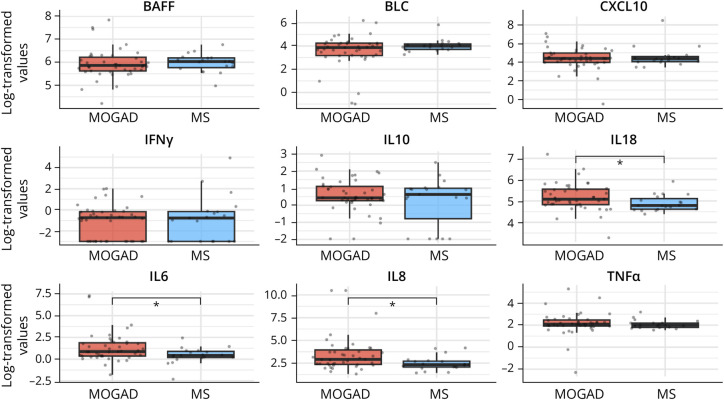
Baseline Serum Cytokines Between Patients With MOGAD and MS With Non–Optic Neuritis Presentations Boxplots depict the distribution of baseline serum log-transformed values of the 9 cytokines between patients with MOGAD and MS with non–optic neuritis presentations. Median values are represented by the horizontal bar, IQR by hinges, 1.5 × IQR by whiskers, and individual values by dots. *p* Values are represented by asterisks as follows: * <0.05. IQR = interquartile range; MOGAD = myelin oligodendrocyte glycoprotein antibody–associated disease; MS = multiple sclerosis.

All the 9 cytokines had comparable baseline serum values between MOGAD and MS cohorts. IL6 values were higher in MOGAD than in MS, but the difference was not statistically significant (*p* = 0.058) (eFigure 1). Within non–optic neuritis (ON) presentations, patients with MOGAD (n = 43) displayed higher median (interquartile range [IQR]) baseline values of IL6 (2.40 pg/mL [1.42–6.60] vs 1.54 [1.21–2.46], *p* = 0.036), IL8 (18.5 [10.7–52.8] vs 10.3 [7.89–15.3], *p* = 0.012), and IL18 (161 [125–258] vs 120 [101–166], *p* = 0.026) compared with patients with MS (n = 19) (Figure 1).

**Figure 2:**
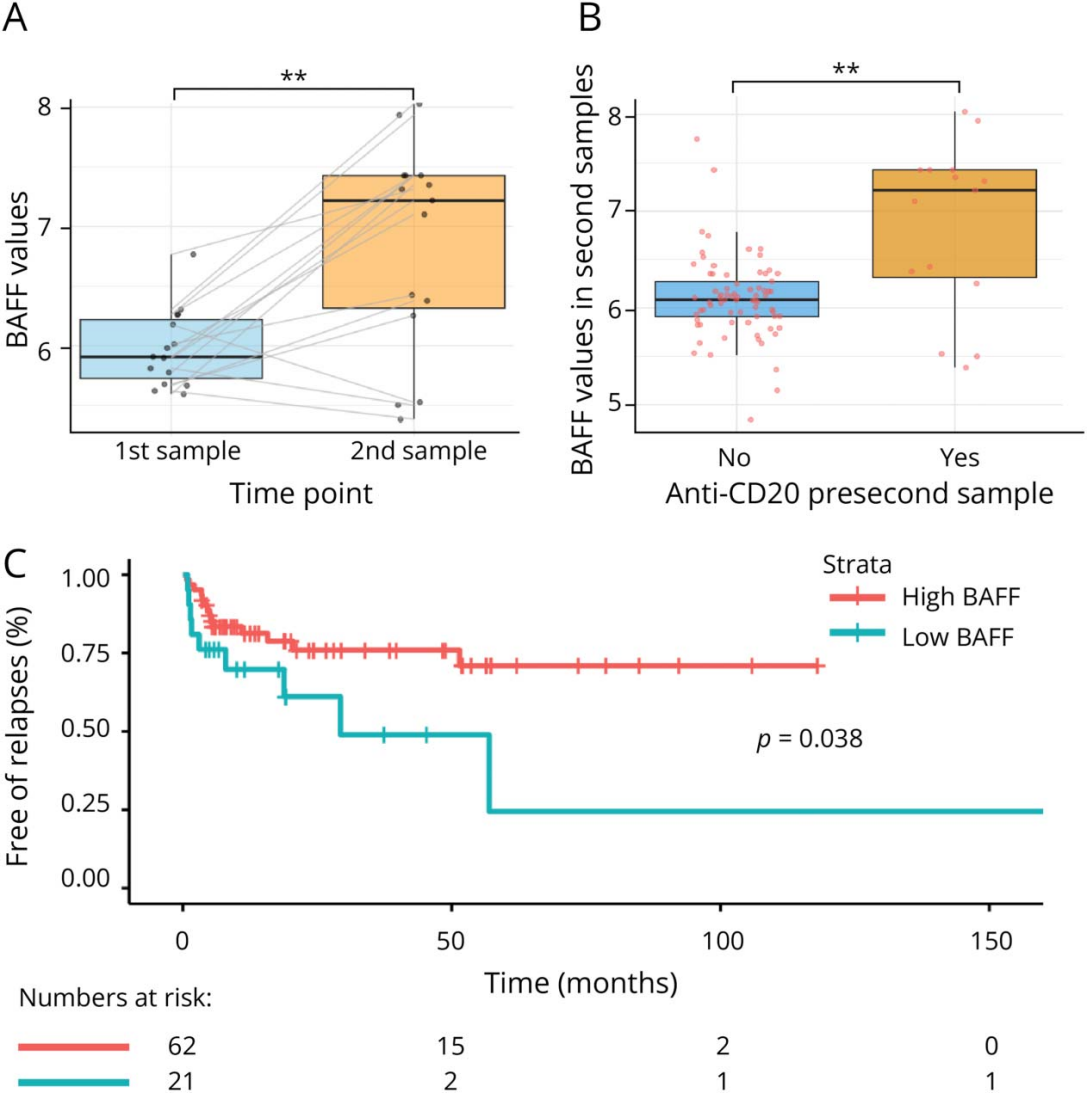
BAFF Increases After Rituximab Treatment and Predicts Relapses During Follow-Up Boxplots depict the distribution of serum BAFF log-transformed values between first and second samples in the 15 patients with MOGAD who received anti-CD20 therapy between both time points (A) and the distribution of serum BAFF log-transformed values in second samples between those patients under anti-CD20 treatment (N = 15) and the remaining patients (N = 73) at the time of second sampling (B). Median values are represented by the horizontal bar, IQR by hinges, 1.5 × IQR by whiskers, and individual values by dots joined by gray lines between time points. *p* Values are represented by asterisks as follows: ** <0.01. (C) The representation of the Kaplan-Meier curves for the time to first relapse between patients with high (≥5.71) and low (<5.71) baseline BAFF values in the MOGAD cohort. The cutoff 5.71 is the 25th percentile value of BAFF in the MOGAD cohort. *Note: five patients were excluded from the Kaplan-Meier and Cox analyses because their baseline samples were obtained after the first relapse. IQR = interquartile range; MOGAD = myelin oligodendrocyte glycoprotein antibody–associated disease.

Regarding cytokine dynamics, BAFF values were increased in the second sample compared with the baseline sample in both MOGAD (*p* = 0.002) and MS (*p* = 0.049) cohorts while the remaining cytokine values were stable. In patients with MOGAD treated with anti-CD20 before second sampling (n = 15), BAFF notably increased in the second sample compared with the first sample (*p* = 0.002) (Figure 2A), but not in the remaining patients (treated with other therapies and nontreated [n = 73]) (*p* = 0.063). Similarly, second samples from patients with MOGAD under anti-CD20 had higher BAFF values compared with the remaining (*p* = 0.002) (Figure 2B).

Baseline BAFF (β 0.06 95% CI [0.01–0.11], *p* = 0.030), CXCL10 (0.10 [0.01–0.20], *p* = 0.036), IL10 (0.11 [0.01– 0.21], *p* = 0.040), and IL8 (−0.21 [−0.41 to −0.02], *p* = 0.033) values were associated with EDSS level at onset. Within non-ON presentations, BAFF (0.08 [0.01–0.16], *p* = 0.047), CXCL10 (0.16 [0.01–0.31], *p* = 0.037), IL10 (0.17 [0.06–0.28], *p* = 0.004), and IL6 (0.14 [0.01–0.28], *p* = 0.046) were associated with EDSS scores at onset. In addition, BAFF values were associated with length of the myelitis on MRI (0.05 [0.01–0.09], *p* = 0.012). No other significant associations were found (eTable 2).

None of the cytokines was associated with a EDSS score ≥ 3.0 at the last follow-up in the whole MOGAD cohort. In non-ON presentations, IL6 (OR 1.51 [1.01–2.54]) and IL8 (1.42 [1.01–2.21]) were associated with this outcome but did not reach statistical significance (*p* = 0.064 and *p* = 0.059, respectively).

Regarding relapses, higher baseline BAFF independently reduced the risk of first relapse after adjustment by proportion of time under chronic treatment (HR 0.41 [0.19–0.89], *p* = 0.024).

Figure 2C shows the Kaplan-Meier survival curve for time to relapse between patients with high and low levels of BAFF (log-rank *p* value = 0.038).

## Discussion

In this multicentric study of adult patients with MOGAD, we conducted a longitudinal analysis of 11 cytokines in serum using an ultrasensitive immunoassay. We confirmed the presence of a proinflammatory Th17-dominant profile in MOGAD with non-ON presentations and the association of cytokines involving different pathways (Th1, Th17, Treg, and B-cell response) with clinical and radiologic severity at onset. The novel and intriguing finding is the association between baseline BAFF levels and risk of relapses during disease course in MOGAD.

Several studies have demonstrated a common profile of cytokines in serum and/or CSF in both MOGAD and AQP4-NMOSD, characterized by an upregulated Th17 (IL6, IL8, IL17) and Treg (IL10) signature, compared with MS, in both adult and pediatric cohorts.^3-5,7-9^ Among them, IL6 has attracted special interest because of its pleiotropic effects such as promoting Th17 cell differentiation, producing autoantibodies by plasmablasts, and increasing the blood-brain barrier permeability.^8^ The approval of satralizumab for AQP4-NMOSD,^2^ with an ongoing trial for patients with MOGAD as well, and the efficacy of tocilizumab in some patients with refractory MOGAD^10^ highlight the relevance of this cytokine. In our cohort, focusing on non-ON presentations, IL6, IL8, and IL18 showed higher baseline levels in MOGAD than in MS. This aligns with some studies reporting higher IL6 levels in non-ON MOGAD phenotypes, especially with brain involvement.^4,8^ In addition, in non-ON MOGAD patients, baseline levels of IL6, BAFF, CXCL10, and IL10 correlated with disease severity at onset, reflecting a more inflammatory component and compensatory regulatory mechanisms in severe presentations. A lesser extent of damage in ON compared with other phenotypes with a less robust inflammatory response could influence the lack of differences in cytokines between MOGAD and MS, and the absence of correlation with clinical status in ON presentations and our total cohort.

Besides the T-cell dominant profile, B cells also play a significant role in MOGAD pathogenesis.^1^ However, few studies have characterized B-cell–related cytokines/chemokines in these patients.^5,7^ Moreover, the possible implication of these molecules on clinical prognosis has not been addressed. In this study, higher baseline values of BAFF predicted lower risk of relapse in patients with MOGAD after adjustment by chronic treatment. This finding provides evidence of the potential protective role of BAFF in MOGAD. In MS, BAFF has shown controversial results.^11,12^ Of interest, a recent study demonstrated that BAFF protects against demyelination and neurodegeneration in an experimental autoimmune encephalomyelitis model and in patients with MS treated with anti-CD20 therapy.^13^ Indeed, blocking BAFF by atacicept in patients with MS led to exacerbated inflammatory disease activity and the interruption of the clinical trial.^14^ In our study, BAFF values increased in patients with MOGAD after anti-CD20 treatment, as reported in several autoimmune diseases, including MS and AQP4-NMOSD.^15^ Whether the BAFF dynamics influence the remarkably different clinic-biological responses to anti-CD20 in MOGAD compared with AQP4-NMOSD and MS^16^ remains unknown.

Some limitations included the retrospective design, the variability in time of follow-up sampling, and the potential influence of treatment especially on cytokine dynamics. Further studies with longer follow-up and larger comparator groups are needed to confirm our results and analyze the association of treatment-dependent BAFF increase with anti-CD20 efficacy in MOGAD.

In conclusion, our results confirm a proinflammatory Th17-dominant profile in non-ON MOGAD patients, with the novel finding of the protective role of baseline BAFF on relapses. These results shed light on the pathogenesis and prognosis of MOGAD, potentially guiding therapeutic decisions.
